# *Bordetella* Type III Secretion Injectosome and Effector Proteins

**DOI:** 10.3389/fcimb.2020.00466

**Published:** 2020-09-04

**Authors:** Jana Kamanova

**Affiliations:** Institute of Microbiology of the Czech Academy of Sciences, Prague, Czechia

**Keywords:** pertussis, *Bordetella*, type III secretion system, effector protein, BteA/BopC, BopN

## Abstract

Pertussis, also known as whooping cough, is a resurging acute respiratory disease of humans primarily caused by the Gram-negative coccobacilli *Bordetella pertussis*, and less commonly by the human-adapted lineage of *B. parapertussis*_HU_. The ovine-adapted lineage of *B. parapertussis*_*OV*_ infects only sheep, while *B. bronchiseptica* causes chronic and often asymptomatic respiratory infections in a broad range of mammals but rarely in humans. A largely overlapping set of virulence factors inflicts the pathogenicity of these bordetellae. Their genomes also harbor a pathogenicity island, named *bsc* locus, that encodes components of the type III secretion injectosome, and adjacent *btr* locus with the type III regulatory proteins. The Bsc injectosome of bordetellae translocates the cytotoxic BteA effector protein, also referred to as BopC, into the cells of the mammalian hosts. While the role of type III secretion activity in the persistent colonization of the lower respiratory tract by *B. bronchiseptica* is well recognized, the functionality of the type III secretion injectosome in *B. pertussis* was overlooked for many years due to the adaptation of laboratory-passaged *B. pertussis* strains. This review highlights the current knowledge of the type III secretion system in the so-called classical *Bordetella* species, comprising *B. pertussis, B. parapertussis*, and *B. bronchiseptica*, and discusses its functional divergence. Comparison with other well-studied bacterial injectosomes, regulation of the type III secretion on the transcriptional and post-transcriptional level, and activities of BteA effector protein and BopN protein, homologous to the type III secretion gatekeepers, are addressed.

## Introduction

The genus *Bordetella* (*Alcaligenaceae*, phylum *Betaproteobacteria*) currently embraces 16 species of the Gram-negative coccobacilli. Its members include important respiratory pathogens of mammals and humans with limited genetic diversity, the so-called classical *Bordetella* species, namely *B. pertussis, B. parapertussis*, and *B. bronchiseptica*. The genus further comprises less extensively studied and phylogenetically distinct *Bordetella* species collectively referred to as non-classical bordetellae, comprising pathogens, opportunistic pathogens, and environmental isolates (Table 1). The phylogenetic analysis of the genus suggests that the animal-associated species likely evolved from their ancestors living in soil and/or water (Hamidou Soumana et al., 2017). The diversification and speciation in the genus were accompanied by the gain and loss of multiple genes, including genes for bacterial protein toxins, protein secretion systems, and other virulence factors (Linz et al., 2016, 2019). The presence of the genes encoding *Bordetella* protein toxins, consisting of adenylate cyclase toxin, pertussis toxin, and dermonecrotic toxin distinguishes classical *Bordetella* species from the non-classical bordetellae (Linz et al., 2016). However, pertussis toxin is solely produced by *B. pertussis* due to mutations in the *ptx* promoter (Arico and Rappuoli, 1987; Parkhill et al., 2003), and dermonecrotic toxin appears to be also imported into *B. avium* that causes respiratory disease of birds called bordetellosis (Linz et al., 2016). For detailed characterization of acquisition and loss of virulence-associated factors during the evolution of the genus *Bordetella* the reader is referred to a recent work of Linz B. and colleagues (Linz et al., 2016). This review aims to explore and discuss the type III secretion system (T3SS) in classical *Bordetella* species, its regulation and mechanism of action, and its role in *Bordetella* infections that is yet to be explored for *B. pertussis*. Remarkably, *bsc-btr* loci encoding *Bordetella* T3SS and its regulatory proteins are also present in *B. ansorpii*, but absent from genomes of all other non-classical bordetellae. A comparison of the genetic organization of *bsc-btr* loci of *B. ansorpii* and classical *Bordetella* species will also be provided.

**Table 1:** Species and lineages of the *Bordetella* genus and their characteristics.

**Species/lineages**	**Host range/Source**	**Disease**	**References**
*B. pertussis*	Humans	Pertussis	Mattoo and Cherry, 2005
*B. parapertussis_*HU*_*	Humans	Pertussis-like disease	Bergfors et al., 1999; Cherry and Seaton, 2012
*B. parapertussis_*OV*_*	Sheep	Asymptomatic chronic infections, pneumonia	Porter et al., 1994
*B. bronchiseptica*	Various mammals	Asymptomatic chronic infections, respiratory disease	Goodnow, 1980; Gueirard et al., 1995; Mattoo and Cherry, 2005
*B. holmesii*	Humans	Pertussis-like disease, septicemia	Weyant et al., 1995; Yih et al., 1999
*B. avium*	Birds	Respiratory disease—bordetellosis	Kersters et al., 1984
*B. hinzii*	Poultry, rabbits, rodents, humans	Opportunistic infections in humans: respiratory disease, septicemia	Vandamme et al., 1995; Register et al., 2015
*B. pseudohinzii*	Rodents	Respiratory tract infections	Ivanov et al., 2016
*B. trematum*	Humans	Opportunistic infections: wound infections, otitis	Vandamme et al., 1996
*B. ansorpii*	Humans	Opportunistic infections: epidermal cyst, blood sample	Ko et al., 2005; Fry et al., 2007
*B. petrii*	Environment, humans	Opportunistic infections: bone infections, respiratory tract infections	Von Wintzingerode et al., 2001; Fry et al., 2005; Le Coustumier et al., 2011
*B. bronchialis*	Humans	Opportunistic infections: respiratory specimen	Vandamme et al., 2015
*B. flabilis*	Humans	Opportunistic infections: respiratory specimen	Vandamme et al., 2015
*B. sputigena*	Humans	Opportunistic infections: respiratory specimen	Vandamme et al., 2015
*B. muralis*	Environment		Tazato et al., 2015
*B. tumulicola*	Environment		Tazato et al., 2015
*B. tumbae*	Environment		Tazato et al., 2015

The human-adapted *B. pertussis* is the primary causative agent of pertussis, also known as whooping cough, a contagious, prolonged respiratory illness that used to be the major cause of infant mortality in the pre-vaccine era (Mattoo and Cherry, 2005). Pertussis remains one of the least controlled vaccine-preventable infectious diseases. In the recent years, an increase in pertussis incidence and/or pertussis outbreaks have been experienced in a number of most developed countries with high vaccine coverage, including the Czech Republic, U.S., U.K., Netherlands, and Australia (Fabianova et al., 2010; Spokes et al., 2010; Burns et al., 2014; Sealey et al., 2016). Unrecognized or mildly symptomatic *B. pertussis* infections in adolescents and adults are common and represent a threat to unvaccinated infants to whom the disease can be fatal (Cherry, 2019). The key contributing factors of increased pertussis incidence are still under debate. Besides greater awareness, improved diagnostics and genetic changes in circulating *B. pertussis* strains, the major cause of pertussis resurgence appears to be the switch from whole-cell pertussis (wP) to the less reactogenic but less effective acellular pertussis (aP) vaccines. These confer significantly shorter-lasting protection and are not efficient in preventing the colonization of the vaccinated individuals by *B. pertussis* and pathogen spread in the population (reviewed in Mooi et al., 2014; Cherry, 2019; Kapil and Merkel, 2019).

Other *Bordetella* species, the human-adapted lineage of *B. parapertussis*_HU_ and the non-classical species *B. holmesii* can also cause pertussis-like disease in humans, although generally accompanied by milder symptoms and shorter illness duration (Bergfors et al., 1999; Yih et al., 1999; Cherry and Seaton, 2012; Rodgers et al., 2013). The ovine-adapted lineage of *B. parapertussis*_*O*__V_ colonizes only sheep with no or little transmission to humans (Van Der Zee et al., 1996). In contrast, *B. bronchiseptica* infects a variety of mammals and causes diverse pathologies that range from typical chronic and often asymptomatic respiratory infections up to more acute diseases, such as the kennel cough in dogs, bronchitis in cats, bronchopneumonia and atrophic rhinitis in piglets, and snuffles in rabbits (Goodnow, 1980; Mattoo and Cherry, 2005). *B. bronchiseptica* infections in humans are rare and occur mostly in immunocompromised patients, children, and in elderly that are in contact with animals (Goodnow, 1980; Gueirard et al., 1995; Mattoo and Cherry, 2005). Nevertheless, clustering of *B. bronchiseptica* strains into two distinct *B. bronchiseptica* subpopulations, complex I, primarily of animal origin (68%), and complex IV that is primarily isolated from humans (80%) was reported based on multilocus sequence typing (MLST), distribution of insertion sequence elements (ISEs) and whole-genome sequence comparisons (Diavatopoulos et al., 2005; Park et al., 2012).

The classical *Bordetella* species are phylogenetically closely related, despite a different range of their mammalian hosts and diverse pathologies they cause. Hence, it was proposed to classify them as subspecies, rather than species (Musser et al., 1986). The *B. pertussis, B. parapertussis*_HU_, and *B. parapertussis*_OV_ likely evolved very recently and independently from different lineages of *B. bronchiseptica*-like ancestors (Van Der Zee et al., 1996, 1997; Parkhill et al., 2003). Specifically, *B. parapertussis*_HU_ and *B. parapertussis*_OV_ appear to have evolved from a *B. bronchiseptica* complex I-like ancestor, whereas *B. pertussis* may have shared a common ancestor with complex IV strains of human-associated lineages of *B. bronchiseptica* (Diavatopoulos et al., 2005; Park et al., 2012). The speciation of *B. pertussis* and *B. parapertussis* has been accompanied by a large-scale gene loss and inactivation. This resulted in a respective reduction of their genome size by 22 and 9%, compared to *B. bronchiseptica* species (cf. *B. pertussis* Tohama I ~ 4.1 Mbp, *B. parapertussis*_HU_ 12822 ~ 4.8 Mbp, *B. parapertussis*_OV_ Bpp5 ~ 4.9 Mbp, *B. bronchiseptica* RB50 ~ 5.3 Mbp). These genomic rearrangements were apparently mediated by the acquisition and expansion of insertion sequence elements (ISEs) and recombination between their copies (Parkhill et al., 2003; Preston et al., 2004; Weigand et al., 2019). Interestingly, host adaptation and speciation of classical *Bordetella* species likely was a consequence of loss of function, rather than a gain of function. As a result, the differences in the virulence of the classical *Bordetella* species are related to the loss of regulatory or control functions and/or sequence polymorphism (Parkhill et al., 2003; Cummings et al., 2004).

## A Brief Overview of the Virulence Factors of Classical Bordetellae

The three classical *Bordetella* species produce a largely overlapping array of virulence factors that are involved in the colonization of the host respiratory tract, immune evasion, and transmission to new hosts. These comprise (i) adhesins, such as filamentous hemagglutinin and fimbriae, (ii) a large number of autotransporters involved in the adhesion and/or resistance to complement, including the adhesion molecule pertactin and complement evasion factor Vag8, and (iii) protein toxins consisting of adenylate cyclase toxin and dermonecrotic toxin (Mattoo et al., 2001; Parkhill et al., 2003; Hovingh et al., 2017). The adenylate cyclase toxin is a potent immunomodulatory toxin that subverts host innate and adaptive immune defenses by its adenyl cyclase activity (reviewed in Fedele et al., 2017) while the dermonecrotic toxin is associated with induction of turbinate atrophy in pigs and appears to have neurotoxic activity (Brockmeier et al., 2002; Teruya et al., 2020). By contrast, pertussis toxin (PTX) that catalyzes the ADP-ribosylation of the alpha subunit of heterotrimeric G proteins of the G_i/o_ class is produced exclusively by *B. pertussis* species. The PTX production is responsible for systemic symptoms of pertussis disease, such as leukocytosis that is associated with the mortality in infants (Pierce et al., 2000; Carbonetti, 2010). The classical *Bordetella* species also differ in the levels of expression of the type III secretion system (T3SS) *in vitro*. Unlike *B. bronchiseptica* and ovine *B. parapertussis*_OV_, the human-adapted *B. pertussis* and *B. parapertussis*_*HU*_ species have the expression of their T3SS blocked at a post-transcriptional level when grown in Stainer-Scholte medium (Mattoo et al., 2004). Therefore, the function of the T3SS in human-adapted *Bordetella* species was overlooked for many years. However, isolates of *B. pertussis* express the T3SS effector protein BteA/BopC (Hegerle et al., 2013). In addition, they produce a functional T3SS upon passage on an infected animal or eukaryotic cells (Fennelly et al., 2008; Gaillard et al., 2011; Bibova et al., 2015), or when grown in media with limiting glutamate and/or iron concentrations (Brickman et al., 2011; Hanawa et al., 2016).

## *Bordetella* Injectosome

**Figure 1:**
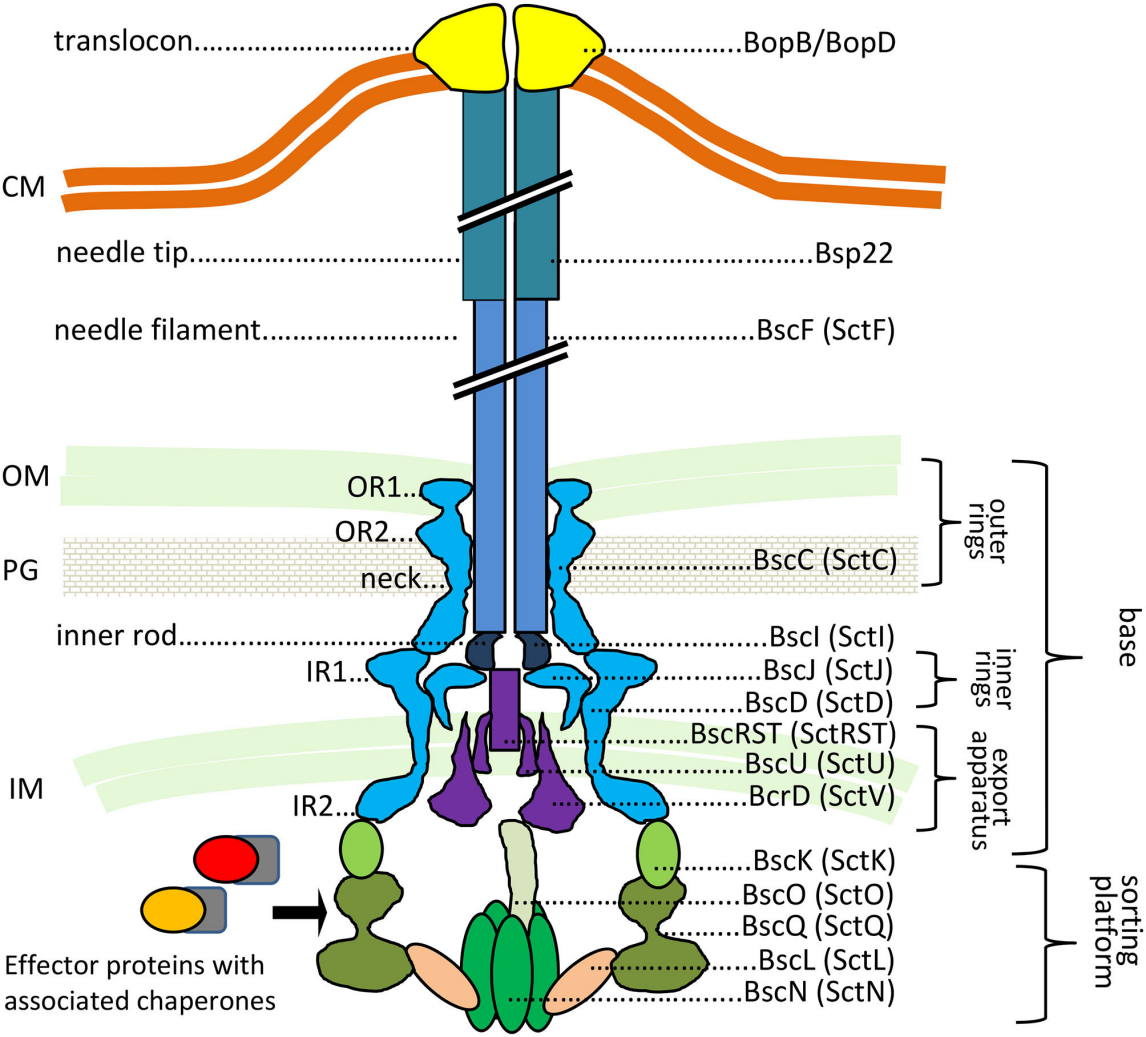
The predicted structure of *Bordetella* injectosome. The diagram is based on Galan et al. (2014), Hu et al. (2017), Park et al. (2018). Known or predicted functions and locations of *Bordetella* proteins are indicated. In *Bordetella* spp., the injectosome needle tip is formed by a helical assembly of the Bsp22 protein (Medhekar et al., 2009). See the text for further details. CM, cytoplasmic membrane; OM, outer membrane; PG, peptidoglycan; IM, inner membrane. The proteins are color-coded based on coding genes shown in Figure 3A.

The T3SS injectosome is a sophisticated protein-export apparatus that enables the delivery of bacterial effector proteins directly from bacterial cytosol into the cytosol of the host cells through a conduit spanning the two bacterial membranes and the plasma membrane of the target cell. It consists of an extracellular needle-like appendage with a central channel of ~2 nm in diameter, which protrudes from the bacterial surface and is linked to a cell wall-embedded secretion system machinery steered by the associated cytoplasmic components, as depicted in Figure 1 (reviewed in Galan et al., 2014; Notti and Stebbins, 2016).

**Figure 2:**
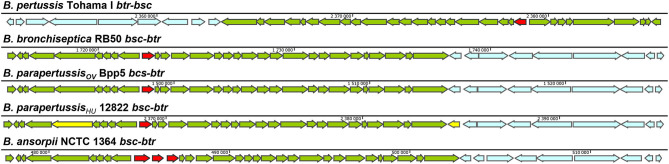
Genetic organization of the type III regulation *btr* and secretion *bsc* loci in different *Bordetella* species. The genetic organization of *btr*-*bsc* loci of *B. pertussis* Tohama I (NC_002929.2, *btr* BP2226—BP2234, *bsc* BP2235—BP2265) and *bsc-btr* loci of *B. bronchiseptica* RB50 (NC_002927.3, *bsc* BB1608-BB1637, *btr* BB1638-BB1646), *B. parapertussis*_OV_ Bpp5 (NC_018828.1, *bsc* BPP5_1370-BPP5_1399, *btr* BPP5_1400-BPP5_1408), *B. parapertussis*_HU_ 12822 (NC_002928.3, *bsc* BPP2211-BPP2240, *btr* BPP2241-BPP2249) and *B. ansorpii* NCTC13364 (FKBS01000014.1, contig: ERS088257SCcontig000014, *bsc* SAMEA1982600_01974-SAMEA1982600_02005, *btr* SAMEA1982600_02006-SAMEA1982600_02014) is shown. The genes of *btr* and *bsc* loci are depicted in blue and green, respectively. The putative pseudogenes BPP2215 and BPP2241 in *bsc-btr* loci of *B. parapertussis*_HU_ 12822 are highlighted in yellow (Parkhill et al., 2003) whereas the Bsp22 tip filament protein gene in *bsc* loci of classical bordetellae and 3 orfs that replaced this gene in *B. ansorpii* are highlighted in red. Arrows indicate the direction of transcription.

The genes for the components of the T3SS injectosome are generally clustered on a mobile genetic element, a pathogenicity island or a plasmid, and appear to have been acquired as an intact genetic block by horizontal gene transfer (Hueck, 1998; Hacker and Kaper, 2000). T3SS injectosomes are widespread among gram-negative bacteria. They are present in not only animal and plant pathogens but also in insect and amoeba pathogens, and are also essential to some symbionts (Troisfontaines and Cornelis, 2005). First genes in *Bordetella* spp. with a high degree of similarity to the genes coding for components of *Yersinia* injectosome were reported by Yuk et al. (1998). In the following years, the whole 22.5 kbp *bsc* (*Bordetella* secretion) locus encoding the *Bordetella* injectosome, and the adjacent 11 kbp *btr* (*Bordetella* type III regulation) locus, encoding injectosome-regulatory proteins, have been described (Kerr et al., 1999; Fauconnier et al., 2001; Mattoo et al., 2004; Ahuja et al., 2016). Genomic analysis of classical bordetellae showed that *Bordetella* injectosome is their most conserved secretion system (Park et al., 2012). The gene positions within the 33.5 kbp *btr-bcs* loci *(btr* BP2226-2234, *bsc* BP2235-BP2265) of *B. pertussis* Tohama I match the organization of the genes in *bsc-btr* loci (*bsc* BB1608-BB1637, *btr* BB1638-BB1646) of *B. bronchiseptica* RB50. In addition, the vast majority of nucleotide substitutions are silent or result in conservative amino acid substitutions, which implies the evolutionary pressure for the preservation of the T3SS in *B. pertussis* (Mattoo et al., 2004). However, compared to *B. bronchiseptica* RB50 genome, the *B. pertussis* Tohama I genomic region harboring the *btr-bsc* underwent inversion, likely due to an ISE-mediated rearrangement (see Figure 2). Interestingly as also depicted in Figure 2, although the *bsc-btr* loci (*bsc* BPP5_1370-BPP5_1399, *btr* BPP5_1400-BPP5_1408) of *B. parapertussis*_OV_ Bpp5 are homologous and intact, the *bsc-btr* loci (*bsc* BPP2211-BPP2240, *btr* BPP2241-BPP2249) of the human-adapted *B. parapertussis*_HU_ 12882 was suggested to contain pseudogenes for regulatory (BPP2241) and structural (BPP2215) proteins (Parkhill et al., 2003; Linz et al., 2016). The functionality of T3SS in human-adapted *B. parapertussis*_HU_ thus remains to be clarified. Remarkably, the *bsc-btr* loci are also present in one non-classical *Bordetella* species, *B. ansorpii* that has so far been isolated only twice (Ko et al., 2005; Fry et al., 2007). The analysis of these loci in *B. ansorpii* NCTC1364 (*bsc* SAMEA1982600_01974-SAMEA1982600_02005, *btr* SAMEA1982600_02006-SAMEA1982600_02014) reveals a homologous organization, suggesting that *B. ansorpii* and classical bordetellae had a common ancestor. However, three different genes (tagged as SAMEA1982600_01983, SAMEA1982600_01984, SAMEA1982600_01985) are present at the position of the gene encoding the Bsp22 tip filament protein of classical bordetellae as highlighted in Figure 2.

**Figure 3:**
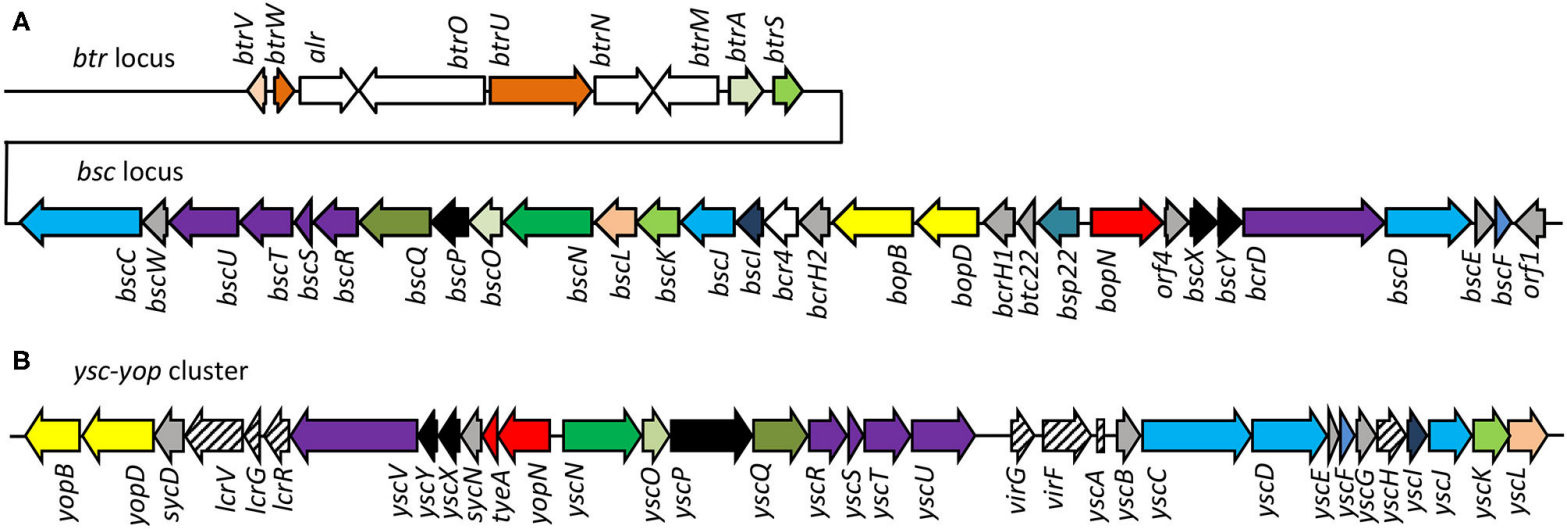
Comparison of genetic organization of *B. pertussis btr-bsc* loci and *Yersinia* Yop-Ysc cluster. **(A)** The type III regulation *btr* and secretion *bsc* loci of the *B. pertussis* Tohama I genome, accession number NC_002929.2, *btr* genes BP2226 – BP2234, *bsc* genes BP2235—BP2265. **(B)** The Yop-Ysc cluster of *Yersinia enterocolitica* plasmid pYVe227, accession number AF102990.1, between *yopB* and *ycL*. Arrows indicate the direction of transcription. Homologies of the encoded injectosome proteins are indicated by the same color code, chaperones are colored in gray.

The injectosome of *Bordetella* spp. encoded in the aforementioned *bsc* locus does not fit comfortably into any of the seven phylogenetic families of the non-flagellar T3SS being classified by loci organization and amino acid sequences of the encoded proteins, and consisting of families Ysc, Inv-Mxi-Spa, Ssa-Esc, Hrc-Hrp 1, Hrc-Hrp 2, the Rhizobiales and the Chlamydiales (Troisfontaines and Cornelis, 2005). The *Bsc* system of *Bordetella* spp. could nevertheless form a subgroup within the Ysc family of injectosomes, which comprises the Ysc system of *Yersinia* spp., Asc system of *Aeromonas* spp., Lsc system of *Photorhabdus luminescens*, Psc system of *Pseudomonas aeruginosa*, and the Vsc system of *Vibrio parahaemolyticus* (Pallen et al., 2005; Troisfontaines and Cornelis, 2005). As depicted in Figures 3A,B, several homologous genes in the *Bordetella bsc* locus (e.g., *bscI* to *bscL*, as well as the *bscN* to *bscU* genes of *Bordetella* spp.) exhibit the same relative positions as the genes of the plasmid-encoded *ysc-yop* cluster of *Y. enterocolitica* W22703. However, the relative positions of other homologous genes differ, with *bscN* immediately following *bscL* in *Bordetella*, while *yscN* is not adjacent to *yscL* in *Y. enterocolitica* (Fauconnier et al., 2001). However, the *bsc*-encoded proteins still show strong sequence similarity to the well-described homologous proteins found in *Yersinia* spp. as listed in Table 2 (Yuk et al., 1998; Kerr et al., 1999; Fauconnier et al., 2001). The precise composition and function of the *Bordetella* injectosome still need to be verified experimentally. Nevertheless, based on studies of the homologous injectosomes of *Yersinia* and *Salmonella* (reviewed in Dewoody et al., 2013; Galan et al., 2014; Galan and Waksman, 2018; Wagner et al., 2018), its structural organization can be predicted with a high degree of confidence to resemble the arrangement depicted in Figure 1.

**Table 2:** *Bordetella bsc*-encoded genes, corresponding proteins, and predicted functions.

**Locus tag *Bp* Tohama I**	**Protein name *Bordetella* spp**.	**Protein name *Yersinia* spp.^**a**^**	**Sct common nomenclature^**b**^**	**Predicted function/functional name**
BP2235	BscC	YscC	SctC	Needle complex outer rings
BP2236	BscW	–	–	T3SS chaperone
BP2237	BscU	YscU	SctU	Export apparatus switch protein
BP2238	BscT	YscT	SctT	Minor export apparatus protein
BP2239	BscS	YscS	SctS	Minor export apparatus protein
BP2240	BscR	YscR	SctR	Minor export apparatus protein
BP2241	BscQ	YscQ	SctQ	C -ring protein
BP2243	BscP	YscP	SctP	Needle length regulator
BP2244	BscO	YscO	SctO	Stalk
BP2245	BscN	YscN	SctN	ATPase
BP2246	BscL	YscL	SctL	Stator
BP2247	BscK	YscK	SctK	Accessory sorting platform protein
BP2248	BscJ	YscJ	SctJ	Needle complex inner rings
BP2249	BscI	YscI	SctI	Inner rod component
BP2250	Bcr4	–	–	?
BP2251	BcrH2	–	–	Class II translocator chaperone
BP2252	BopB	YopB	–	Effector translocator, pore protein
BP2253	BopD	YopD	–	Effector translocator, pore protein
BP2254	BcrH1	–	–	Class II translocator chaperone
BP2255	Btc22 (Orf6)^c^	–	–	Bsp22 chaperone
BP2256	Bsp22	–	–	Tip filament protein
BP2257	BopN	YopN / TyeA	SctW	Gatekeeper
BP2258	Orf4	–	–	T3SS chaperone
BP2259	BscX (Orf3)^d^	YscX	–	T3SS protein X
BP2260	BscY (Orf2)^d^	YscY	–	T3SS protein Y
BP2261	BcrD	YscV (LcrD)	SctV	Major export apparatus protein
BP2262	BscD	YscD	SctD	Needle complex inner rings
BP2263	BscE	–	–	T3SS chaperone
BP2264	BscF	YscF	SctF	Needle filament protein
BP2265	Orf1	–	–	T3SS chaperone

a Homologues from Yersinia spp. T3SS are given for reference;

b Where applicable, the universal nomenclature for T3SS structural proteins is listed to allow for comparison; -, not present;

c (Kurushima et al., 2012a);

d *(Gurung et al., 2018)*.

By analogy, the base or the so-called basal body of *Bordetella* injectosome, embedded in the bacterial envelope, would consist of two membrane-spanning ring structures. The outer membrane ring would be built by oligomerization of BscC and the two concentric inner membrane rings would be formed by oligomerization of BscD on the outside and BscJ on the inside. The basal body would connect with the inner membrane export apparatus, formed by the BscRSTU and BcrD (SctV homolog) components. The latter would interact with the cytosolic sorting platform and additional regulatory proteins to allow for hierarchy in protein secretion, substrate unfolding, and export. By analogy to *Salmonella*, the sorting platform would be composed of three scaffolding proteins BscK, BscQ, BscL, and an ATPase BscN, linked to the export apparatus through another component, BscO (Hu et al., 2017). The needle filament composed of polymerized BscF would then attach to the basal body through the inner rod made from the BscI protein, and extend into the extracellular milieu forming a rigid hollow conduit for the secretion of proteins (Figure 1). The *Bordetella* needle filament on its distal end appears to be capped by another hollow helical assembly composed of the filament tip protein Bsp22 (Medhekar et al., 2009). The Bsp22 protein undergoes spontaneous polymerization and requires its chaperone Btc22 (formerly Orf6) for stabilization in the bacterial cytoplasm (Kurushima et al., 2012a; Villarino Romero et al., 2013). Bsp22 also binds directly to BopD, a component of the *Bordetella* translocon pore and it is essential for effector protein delivery into the target cells (Medhekar et al., 2009). It is thus assumed that the Bsp22 polymer forms a long flexible connecting channel that links the needle filament to the translocon pore inserted within the target cell membrane, as depicted in Figure 1. Indeed, similar needle extensions were described to be formed by the filament tip protein EspA of enteropathogenic *Escherichia coli* (EPEC) (Daniell et al., 2001; Sekiya et al., 2001; Wang et al., 2006). Intriguingly, the EspA filament appears to be eliminated upon attachment of EPEC to target cells (Knutton et al., 1998) and the function of the Bsp22 and EspA-formed needle extensions is currently unknown. It has been speculated that EspA filaments may cross the barrier of the mucous layer and help in adhesion to epithelial cells and/or in biofilm formation (Daniell et al., 2001; Cleary et al., 2004; Moreira et al., 2006). The *Bordetella* proteins BopD and BopB hetero-oligomerize with unknown stochiometry within the host plasma membrane and form the translocon pore that functions as the conduit for translocation of T3SS effector proteins into host cell cytosol (Kuwae et al., 2003; Nogawa et al., 2004), as depicted in Figure 1. Both BopD and BopB proteins are required for the pore-forming hemolytic activity of the injectosome on red blood cells but are not needed for *in vitro* secretion of other T3SS substrates (Kuwae et al., 2003; Nogawa et al., 2004).

Additional regulatory and structural components, presumably involved in the timing of protein secretion by the injectosome, are encoded within the *Bordetella bsc* locus. These include proteins BscX (formerly Orf3, homologous to T3SS protein X) and BscY (formerly Orf2, homologous to T3SS protein Y), BscP, and BopN (Table 2). The BscX and BscY proteins would by analogy with *Yersinia* YscX and YscY orchestrate the secretion of early substrates through their interaction with BcrD (Diepold et al., 2012). The YscX- and YscY-like proteins are unique to the Ysc family of injectosomes and are not encoded within other injectosome families (Gurung et al., 2018). BscP would by analogy with SctP protein control the length of BscF needle filament by a poorly understood mechanism (reviewed in Diepold and Wagner, 2014). Finally, the BopN protein would activate effector protein secretion upon contact with the host cell, in the so-called “second substrate switching event,” as deduced from its homology to the gatekeeper protein, SctW (reviewed in Portaliou et al., 2016). The BopN function in *Bordetella* injectosome, however, remains unclear and will be discussed in the section on effector proteins. The *bsc* locus further encodes the chaperones for the respective components of the injectosome (Table 2), e.g., Bsp22 chaperone Btc22 (formerly Orf6), the putative chaperone BcrH2 that co-immunoprecipitates with the BopB-BopD complex from bacterial cytosol, and one additional protein, called Bcr4, with unclear activity (Nogawa et al., 2004; Kurushima et al., 2012a; Nishimura et al., 2018).

## Type III Secretion Regulation in *Bordetella*

**Figure 4:**
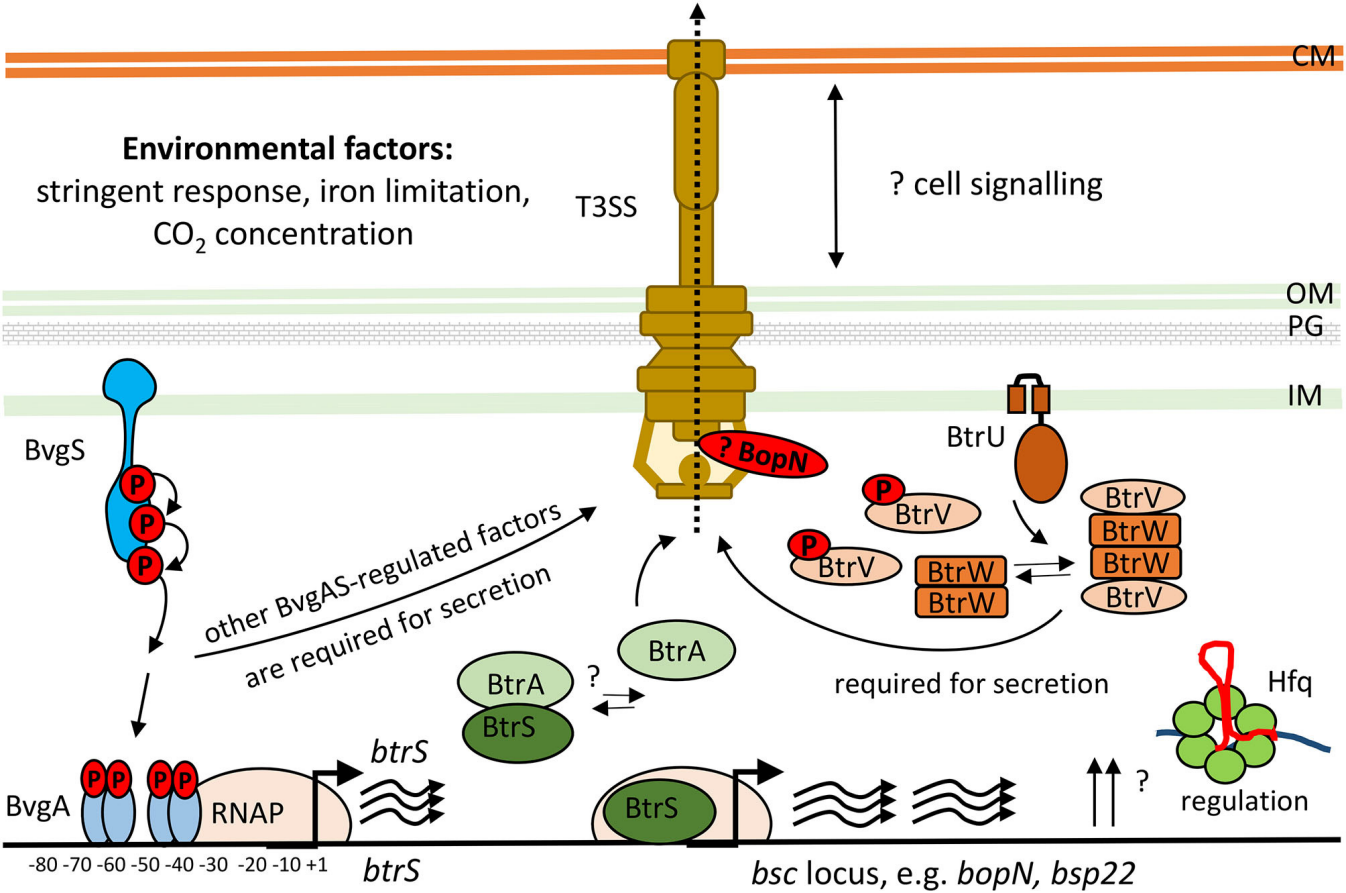
Scheme of transcriptional and post-transcriptional regulation of T3SS in *Bordetella* species. The two-component system, BvgAS, composed of the sensor kinase BvgS and the response regulator BvgA activates expression of the extracytoplasmic function (ECF) sigma factor BtrS and regulates additional factors necessary for T3SS function (Mattoo et al., 2004; Moon et al., 2017). The activity of BtrS leads to transcription of several *bsc*-encoded genes while being counteracted by the T3SS-exported anti-sigma factor, BtrA (Mattoo et al., 2004; Kurushima et al., 2012c; Ahuja et al., 2016). See text for further details. Besides, the partner-switcher proteins BtrU, BtrW, and BtrV control secretion through the injectosome via a series of serine phosphorylation and dephosphorylation events by a yet unknown mechanism (Mattoo et al., 2004; Kozak et al., 2005). BopN protein homologous to the gatekeeper StcW might also regulate the secretion post-transcriptionally. Also RNA chaperone Hfq is required for the expression of T3SS genes in *B. pertussis* (Bibova et al., 2015; Dienstbier et al., 2019). CM, cytoplasmic membrane; OM, outer membrane; PG, peptidoglycan; IM, inner membrane.

The *bsc*-encoded genes of the *Bordetella* injectosome are induced during infection, and are responsive to blood or serum exposure, and increased CO_2_ concentrations (Gaillard et al., 2011; Hester et al., 2012; Bibova et al., 2015; Gestal et al., 2018; Van Beek et al., 2018; Wong et al., 2019). Other stimuli that can activate T3SS expression and secretion in bordetellae are the stringent response induced by iron limitation and/or starvation for carbon source, as depicted in Figure 4 (Brickman et al., 2011; Kurushima et al., 2012b; Hanawa et al., 2016). However, upon the internalization of bordetellae into macrophages the transcription of injectosome genes is down-regulated (Rivera et al., 2019; Petrackova et al., 2020). The *bsc*-encoded genes are under the control of *Bordetella* master virulence regulatory system BvgAS. This two-component system is composed of the membrane-bound sensor kinase BvgS and of its phosphorylation substrate, the DNA-binding response regulator protein BvgA, which coordinates expression of hundreds of genes (Hot et al., 2003; Cummings et al., 2006; Nicholson, 2007; Moon et al., 2017). The system appears to function as a “rheostat” controlling a spectrum of phenotypic modes in response to environmental clues. Nevertheless, the exact nature of the signals and the mechanism by which these signals are perceived and integrated into the BvgAS regulon remains unknown (reviewed in Mattoo et al., 2001; Chen and Stibitz, 2019).

The BvgA-mediated activation of injectosome genes occurs at least for part of them indirectly through an extracytoplasmic function (ECF) sigma factor BtrS, annotated as BrpL, which is encoded in the *btr* locus (Figure 3A, Table 3) (Mattoo et al., 2004; Moon et al., 2017). As shown in Figure 4, in the BvgS-active mode, the membrane-bound phosphorelay sensor kinase BvgS phosphorylates the transcriptional activator BvgA, which in turn directly activates *btrS* transcription by binding as head-to-head BvgA dimers at positions centered at −41.5 and −63.5 upstream of the transcriptional start site (Moon et al., 2017). The BtrS can then activate transcription of *bsp22* and *bopN* genes encoded in the *bsc* locus (Mattoo et al., 2004). However, the ectopic expression of *btrS* in the Bvg^−^ locked mutant of *B. bronchiseptica* RB50 strain (Δ*bvgS*) does not allow for T3SS secretion. Additional unknown Bvg^+^ factors are thus required for the secretion process (Mattoo et al., 2004). Interestingly, two differently regulated gene clusters in the *bsc* locus of *B. bronchiseptica* RB50 strain were identified. The first cluster required the presence of BtrS for transcription (*bscN* to *bsp22*, and *bopN* to *orf2)* whereas genes in the other cluster (*bscC* to *bscO*, and *bcrD* to *bscF*) showed very little dependence, if any, on the presence of BtrS (Ahuja et al., 2016).

**Table 3:** *Bordetella btr*-encoded genes, corresponding proteins, and functions.

**Locus tag *Bp* Tohama I**	**Protein name**	**Functional name**	**Function**	**References**
BP2226	BtrV	STAS domain-containing protein (Anti-sigma factor antagonist)	T3SS regulator	Mattoo et al., 2004
BP2227	BtrW	Serine kinase	T3SS regulator	Mattoo et al., 2004
BP2228	alr	Alanine racemase	unknown	–
BP2229	BtrO	MFS transporter	unknown	–
BP2230	BtrU	Serine phosphatase, SpoIIE family	T3SS regulator	Mattoo et al., 2004
BP2231	BtrN	ABC transporter	unknown	–
BP2232	BtrM	Gamma-glutamylcysteine synthetase	unknown	–
BP2233	BtrA (BspR)	Secreted anti-ECF sigma factor	Binds BtrS, T3SS inhibitor	Kurushima et al., 2012c; Ahuja et al., 2016
BP2234	BtrS (BrpL)	ECF sigma factor	T3SS activator	Mattoo et al., 2004; Ahuja et al., 2016

Besides of the ECF sigma factor BtrS, which controls also other regulatory networks (Gestal et al., 2019), additional genes encoded in the *btr* locus are linked to the injectosome function. These comprise the anti-sigma factor BtrA and the partner-switcher proteins BtrU, BtrW, and BtrV, respectively (Figure 3A, Table 3). As depicted in Figure 4, the secreted anti-sigma factor BtrA, also called BspR (*Bordetella* secreted protein regulator) was suggested to function as a secreted BtrS antagonist and establish a positive feedback loop that couples the injectosome secretion with the expression of T3SS genes in both *B. pertussis* and *B. bronchiseptica* species (Ahuja et al., 2016). The deletion of BtrA, indeed, enhances T3SS-dependent secretion and tissue culture phenotypes of both bacterial species (Kurushima et al., 2012c; Ahuja et al., 2016), and differential control over BtrA intracellular levels was suggested to contribute to the distinct T3SS activities of *B. pertussis* and *B. bronchiseptica* (Ahuja et al., 2016). However, the exact mechanism of BtrA-mediated inhibition of T3SS expression in *Bordetella* species remains to fully clarified. The work of Ahuja and colleagues used yeast two-hybrid system to show that BtrA interacts with BtrS, however, Kurushima and colleagues failed to detect this interaction using GST-pull down assay (Kurushima et al., 2012c; Ahuja et al., 2016).

Although studied so far only in *B. bronchiseptica*, the cascade that regulates T3SS secretion in bordetellae likely involves the partner-switcher regulatory proteins BtrU, BtrW, and BtrV (Figure 4, Table 3). These proteins exhibit homologies to partner-switching complexes of other bacteria that consist of a phosphatase, homologous to BtrU, a protein kinase/anti-sigma factor, homologous to BtrW, and an antagonist protein/anti-anti-sigma factor, homologous to BtrV. Nevertheless, the precise mechanism of regulation in *Bordetella* seems to differ from other bacteria, as all of the BtrU, BtrW, and BtrV proteins are required for *Bordetella* T3SS secretion and none of them acts as a negative regulator. BtrV seems to exert post-transcriptional control required for translation and/or protein stability, whereas BtrU and BtrW are assumed to specifically govern the secretion process (Mattoo et al., 2004; Kozak et al., 2005). Another level of complexity of regulation of T3SS production comes from the post-transcriptional regulator Hfq, a small hexameric RNA-binding protein (reviewed in Chao and Vogel, 2010). Indeed, the Hfq chaperone that anneals small RNA (sRNA) molecules to mRNA targets was found to be required for the expression of some of the T3SS genes (Figure 4) (Bibova et al., 2015; Dienstbier et al., 2019).

## Effector Proteins of *Bordetella* Injectosome

The function of the T3SS injectosome consists in the transport of bacterial effector proteins into the host cell, where these modulate host cell functions by diverse molecular activities for the benefit of the bacteria. Only two effector proteins were so far reported to be present in classical bordetellae, namely the effector protein BteA, also called BopC, and the BopN protein, a homolog of a T3SS regulator (Panina et al., 2005; Kuwae et al., 2006; Nagamatsu et al., 2009).

The BteA effector protein was originally identified by Panina EM and colleagues in 2005 using a computational screen for chaperone-effector loci in *B. bronchiseptica* (Panina et al., 2005). Although the chaperone-effector pair, designated *btcA*-*bteA*, is located 2.5 Mbp away from the *bsc* locus, *bteA* expression is coordinated with the expression of injectosome genes and is activated by the BvgAS system and ECF sigma factor BtrS (Panina et al., 2005; Ahuja et al., 2016). As expected, BteA secretion depends on the T3SS ATPase BscN, and also on the BtrU, BtrW, BtrV partner-switcher proteins (Panina et al., 2005). Upon translocation into the host cells, BteA of *B. bronchiseptica* (*Bb* BteA) induces potent cytotoxicity. Compared to the wild type strains, inducing caspase 1-independent necrotic cell death, *bteA-*deficient strains of *B. bronchiseptica* exhibit negligible cytotoxicity levels similar to the type III secretion-deficient Δ*bscN* strains (Stockbauer et al., 2003; Panina et al., 2005; Kuwae et al., 2006; Ahuja et al., 2012). The *Bb* BteA effector protein alone is capable of inducing potent cytotoxicity in tissue culture and also yeast cells since even trace amounts of *Bb* BteA (undetectable by fluorescence microscopy or Western blot) are cytotoxic (Panina, 2007, dissertation thesis; French et al., 2009).

**Figure 5:**
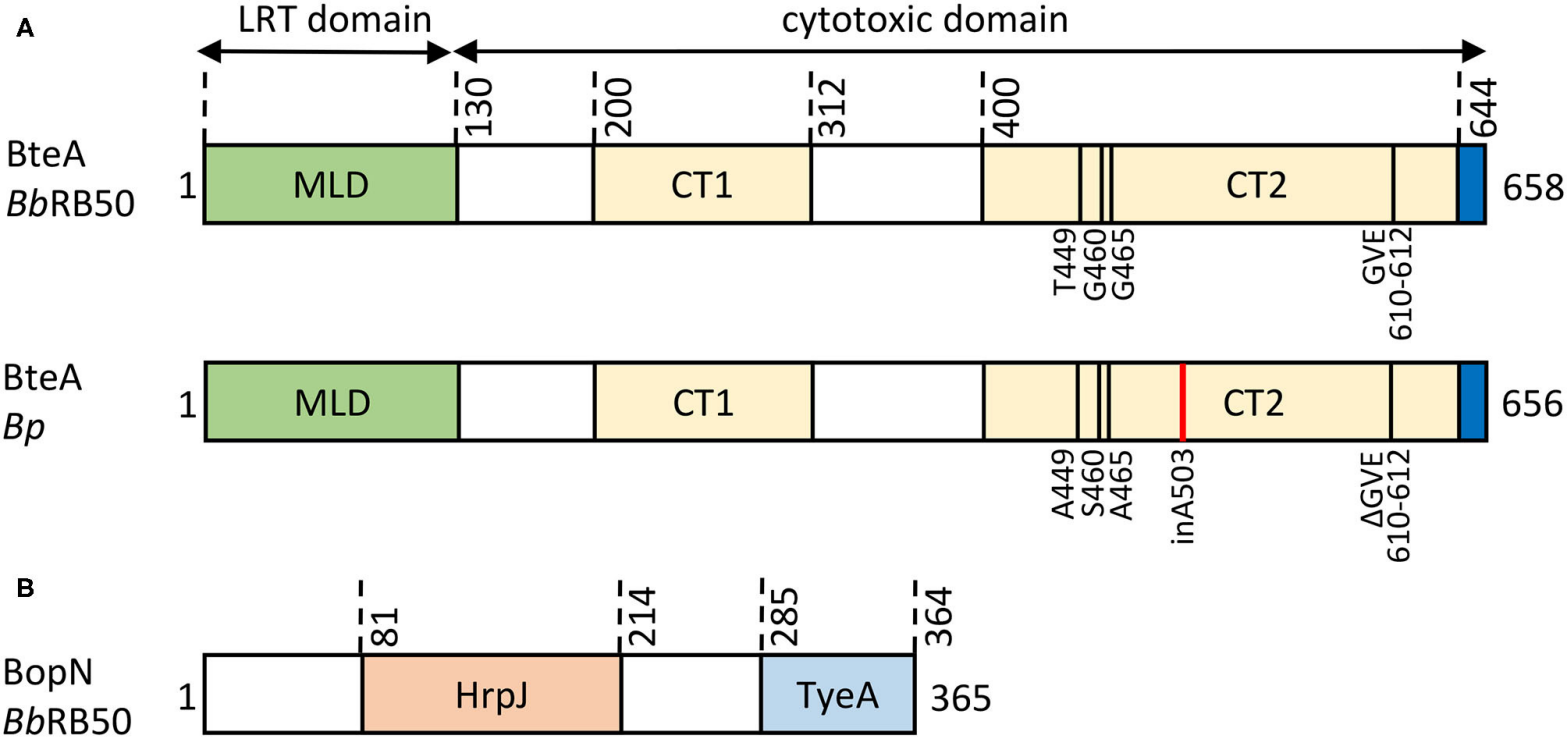
Structural organization of *Bordetella* T3SS effector proteins. **(A)** Schematic representation of the BteA effector protein of *B. bronchiseptica* RB50 (*Bb*RB50 BteA, 658 aa) and *B. pertussis* Tohama I (*Bp* BteA, 656 aa). BteA is composed of two primary domains: a lipid-raft targeting domain (LRT) and a cytotoxic domain. The N-terminal LRT domain of ~ 130 residues is a multifunctional four helical bundle membrane localization domain (MLB) that harbors the secretion signal, comprises BtcA chaperone binding sites, and binds phosphatidylinositol 4,5-bisphosphate (PIP_2_) (French et al., 2009; Guttman et al., 2013; Yahalom et al., 2019). The C-terminal cytotoxic domain of ~ 528 aa contains 2 segments associated with cytotoxicity (*Bb*RB50 BteA, CT1, aa 200-312, CT2, aa 400-658; *Bp* BteA, CT1, aa 200-312, CT2, aa 400-656) (Kuwae et al., 2016). In addition, the last 14 aa were shown to be critical for BteA cytotoxicity (French et al., 2009). Differences in the primary structure of *Bb*RB50 BteA and *Bp* BteA are shown, insertion of A503 within *Bp* BteA is highlighted in red. **(B)** Schematic representation of the 365 residue-long BopN protein of *B. bronchiseptica* RB50 (*Bb*RB50 BopN). The HrpJ (aa 81-214) and TyeA (aa 285-364) domains are indicated.

The 69 kDa BteA effector protein exhibits a modular architecture and is composed of an N-terminal multifunctional lipid raft targeting domain (LRT) of ~ 130 amino acid residues, and a cytotoxic C-terminal domain of ~528 amino acid residues, as depicted in Figure 5A. The LRT domain is rich in highly hydrophobic (~20% are Ile, Leu, Val) and positively charged (>10% are Arg, Lys) amino acid residues, resembling other known membrane localization domains (MLD) but targeting a specific portion of the plasma membrane. Therefore, the membrane localization domain of BteA was called the lipid raft targeting (LRT) domain (reviewed in Geissler, 2012). Within bacteria, the LRT domain binds the cognate chaperone BtcA that guides BteA for injectosome secretion upon recognition of the N-terminal secretion signal of the LRT (Panina et al., 2005; Kuwae et al., 2006). It is assumed that BtcA-BteA complex has a stoichiometry of 2:1 (chaperon:effector) like the chaperon-effector pair, InvB-SipA (Lilic et al., 2006; Guttman et al., 2013). Upon T3SS-mediated translocation into target cell cytosol, the LRT appears to mediate BteA localization into the cytosolic leaflet of lipid rafts of cell plasma membrane via phosphatidylinositol 4,5-bisphosphate (PIP2) binding (French et al., 2009; Yahalom et al., 2019). The crystal structure shows that LRT is an elongated four-helix bundle packed against two shorter perpendicular helices, the second of which caps the domain in a tip motif. The continuous positively charged surface of the second bundle helix was proposed to mediate a direct electrostatic interaction with the negatively charged PIP2 head while being supported by the structural tip helix (Yahalom et al., 2019). Interestingly, homologous domains responsible for lipid raft targeting, but surrounded by other domains, are also present in several known and predicted T3SS effectors and MARTX (multifunctional autoprocessing repeats-in-toxin) toxins, including Plu4750 and Plu3217 from *Photorhabdus luminescens*. The membrane-localization LRT domain thus seems to have been reshuffled during evolution (Panina et al., 2005; French et al., 2009). The C-terminal domain of BteA of ~ 528 amino acid residues (Figure 5A) is solely responsible for the BteA-mediated cytotoxicity (French et al., 2009; Kuwae et al., 2016). Whereas no reliable predictions of the mechanism of BteA-mediated cytotoxicity can be obtained by sequence homology searches, the deletion mutagenesis suggests that the cytotoxic domain of BteA harbors two separate cytotoxic activity-related regions that span over the amino acid residues 200–312 and 400–658, respectively (Kuwae et al., 2016). The last 14 amino acid residues of BteA are also critical for full cytotoxic activity of BteA (French et al., 2009). However, the mechanism underlying the cytotoxic action of BteA as well as its cellular targets remain unknown. The targets of BteA action were proposed to be associated with the cholesterol-rich domains of the host cell membrane since depletion of membrane cholesterol protected cells from the T3SS-dependent cytotoxic action of BteA (French et al., 2009). Nevertheless, the observed protection could have also been due to diminished translocation of BteA into cholesterol-depleted cells (Hayward et al., 2005). Besides, the previously reported T3SS-mediated dephosphorylation of tyrosine residues of proteins in infected mammalian cells appears to be a rather indirect consequence of the cytotoxic action of BteA (Yuk et al., 1998; Kuwae et al., 2006).

The BteA effector proteins of classical *Bordetella* species were claimed to be functionally interchangeable (French et al., 2009). However, our recent study by Bayram and colleagues demonstrated that compared to its BteA homolog from the *B. bronchiseptica*, the BteA effector of *B. pertussis* (*Bp* BteA) exhibits a significantly reduced specific cytotoxic activity toward cultured cells (Bayram et al., 2020). This activity difference could be unambiguously attributed to the insertion of a single alanine residue at position 503 of the *Bp* BteA protein (Figure 5A). Indeed, the specific cytotoxic activity of the *Bp* BteA protein was strongly increased upon deletion of the A503 residue, and the activity of the *Bb* BteA protein was strongly reduced by insertion of an A503 residue, respectively (Bayram et al., 2020). This explains why low cytotoxicity was observed in cells infected by *B. pertussis* translocating BteA through a functional T3SS injectosome (Han et al., 2011; Ahuja et al., 2016). Remarkably, the analysis of amino acid sequences of BteA of the classical bordetellae revealed that the A503 residue is conserved across all *B. pertussis* lineages but is absent in the BteA of all distinct subpopulations and lineages of *B. bronchiseptica* and *B. parapertussis* species. This suggests that the acquisition of the A503 residue in the *Bp* BteA protein occurred early in the *B. pertussis* speciation to human hosts (Bayram et al., 2020). Interestingly, *B. ansorpii* does not appear to encode BteA effector protein homolog nor *Bordetella* master virulence regulatory system BvgAS.

The BopN protein was originally identified as a *Bordetella* T3SS-secreted protein that exhibits homology to the gatekeeper proteins, StcW (Table 2) (Yuk et al., 2000). Remarkably, in the Ysc family of injectosomes, the StcW protein is encoded as two polypeptides, e.g., YopN and its C-terminus-binding chaperone TyeA. There is a single protein corresponding to a chimeric product of YopN-TyeA in the other T3SS systems, including classical *Bordetella* species. The 39 kDa BopN protein (Figure 5B) thus highlights the divergence of *Bordetella* Bsc system away from the Ysc family of injectosomes (Pallen et al., 2005). The function of the StcW is to regulate translocator secretion and/or prevent a premature secretion of the effector proteins presumably through binding to the export apparatus in a protein complex that is released upon activating signal (sensing of the contact to a host cell) (Schubot et al., 2005; Portaliou et al., 2017; Yu et al., 2018). The SctW protein is then either secreted as is the case of *Yersinia* YopN and *Shigella* MxiC, or is degraded like the *Salmonella* SPI-II SsaL protein (Cheng et al., 2001; Botteaux et al., 2009; Yu et al., 2010). The fate of SctW protein if injected into the target host cell is not clear. It is assumed that the SctW does not exert any effector function, except for the *Chlamydia* CopN protein that was shown to induce G2/M cell cycle arrest by inhibiting tubulin polymerization (Huang et al., 2008; Archuleta et al., 2011; Nawrotek et al., 2014).

The function of the *Bordetella* BopN protein remains controversial (Figure 4). The protein was reported to be required for manifestation of the full BteA-mediated cytotoxicity in *B. bronchiseptica* infections of rat L2 pulmonary epithelial cells but was not required for cytotoxicity in mouse DC2.4 dendritic cells (Nagamatsu et al., 2009; Abe et al., 2017). Besides, the deletion of BopN did not affect the secretion of BteA into the culture supernatants of *B. bronchiseptica* grown *in vitro* (Abe et al., 2017). This would indicate that effector secretion in *B. bronchiseptica* does not require host cell contact and/or gatekeeper function, or that the used culture medium artificially mimics the contact to a cell, as previously observed for other T3SS-expressing bacterial species, e.g., enteropathogenic *Escherichia coli* (EPEC), *Vibrio parahaemolyticus* or *Shigella flexnerii* (Deng et al., 2005; Botteaux et al., 2009; Tandhavanant et al., 2018). Remarkably, the BopN protein was reported to be translocated into cells where it was suggested to localize to the cell nucleus (Nagamatsu et al., 2009; Abe et al., 2017). Its activity was also proposed to down-regulate MAPK signaling, block nuclear translocation of the NF-kBp65 subunit while promoting translocation of the NF-kBp50 subunit, and enhancing IL-10 production (Nagamatsu et al., 2009). However, the molecular basis of these processes remain unknown.

## Type III Secretion in Infections With Bordetellae

**Figure 6:**
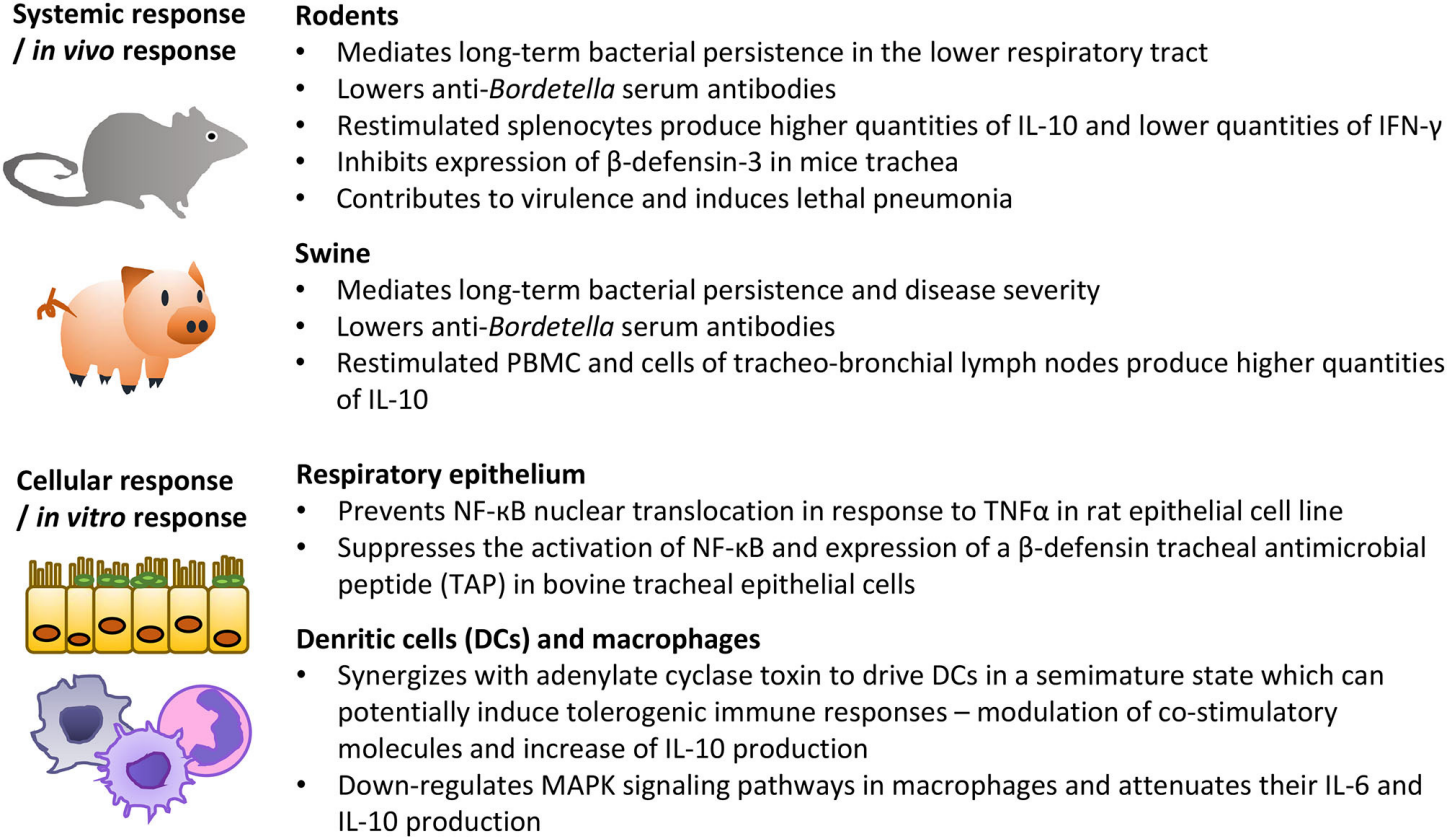
Summary of the most important activities of *B. bronchiseptica* T3SS in animal models *in vivo* and on cells *in vitro*. See the text for further details and references.

The role of T3SS-mediated delivery of effector proteins is thoroughly characterized in animal infections with *B. bronchiseptica*. The elimination of T3SS function due to the deletion of the T3SS ATPse BscN results in defects of *B. bronchiseptica* persistence in the lower respiratory tract of rats, mice, and pigs (Figure 6) (Yuk et al., 1998, 2000; Nicholson et al., 2014). The functional T3SS of *B. bronchiseptica* was also reported to lower titres of anti-*Bordetella* serum antibodies and inhibit the generation of *IFN-*γ-producing splenocytes while enhancing the production of immunosuppressive IL-10 (Figure 6) (Yuk et al., 2000; Skinner et al., 2005; Pilione and Harvill, 2006; Nicholson et al., 2014). Since natural clearance of *B. bronchiseptica* depends on antibodies and production of IFN-γ while IL-10 promotes bacterial colonization, these results suggest that T3SS activity could favor bacterial persistence by altering the balance between IL-10 and IFN-γ, and hindering the antibody production (Kirimanjeswara et al., 2003; Skinner et al., 2005; Pilione and Harvill, 2006). Nevertheless, it appears that T3SS primarily targets innate immunity functions since Δ*bscN* strain was found to be hypervirulent in SCID-beige mice that are devoid of functional B cells and T cells (SCID) and NK cells (beige mutation) (Yuk et al., 2000). The T3SS may synergize with adenylate cyclase toxin to modulate macrophage and dendritic cell phenotypes and thereby subvert adaptive immune responses (Figure 6) (Skinner et al., 2004, 2005; Reissinger et al., 2005; Siciliano et al., 2006). Alternatively, or in combination, T3SS action may consist in inhibition of NF-kB activation in epithelial cells of the respiratory epithelia (Figure 6) (Yuk et al., 2000; Legarda et al., 2005; Ryan et al., 2018). Indeed, infection with wild type *B. bronchiseptica*, but not with the Δ*bscN* mutant, suppressed the activation of NF-κB and induction of β-defensin in primary bovine tracheal epithelial cells and/or during mice infection (Legarda et al., 2005; Ryan et al., 2018). Interestingly, though, T3SS-dependent phenotypes of *B. bronchiseptica* may vary between different phylogenetic lineages and/or isolates (Figure 6) (Buboltz et al., 2009; Ahuja et al., 2012). For example, enhanced expression of T3SS genes was reported to be partially responsible for the increased virulence of complex I *B. bronchiseptica* 1289 strain isolated from diseased host, as compared to the virulence of the RB50 strain isolated from an asymptomatic host (Cotter and Miller, 1994; Buboltz et al., 2009). Along the same line, *in vitro* T3SS-mediated hyper-cytotoxicity of a subset of complex IV *B. bronchiseptica* strains correlated with the increased ability of these strains to cause lethal pulmonary infections in mice (Ahuja et al., 2012).

The potential role of T3SS in *B. pertussis* infections was overlooked for many years due to adaptional block of injectosome expression in *B. pertussis* until the seminal studies of Fennelly et al. (2008); Gaillard et al. (2011). The activity of *B. pertussis* T3SS was then suggested to dampen the inflammatory responses in infected mice by inhibiting proinflammatory cytokine production and enhancing anti-inflammatory IL-10 production in the lungs early after infection, which correlated with lower antigen-specific IFN-γ, IL-17 and IgG responses later in the infection (Fennelly et al., 2008). These data, however, warrant further investigation. Surprisingly, compared with wild type bacteria, the T3SS-deficient *B. pertussis* mutant was significantly impaired in the capacity to colonize the lungs already 3 h after respiratory challenge (Fennelly et al., 2008).

The functionality of *B. pertussis* T3SS injectosome is intriguing, given the acute nature of pertussis disease, which is relatively short-lived. This contrasts with the rather chronic infections caused by *B. bronchiseptica*, which have been related to the action of the T3SS (Yuk et al., 1998, 2000; Nicholson et al., 2014) and/or BteA effector protein (Panina, 2007, dissertation thesis). The adaptation of T3SS of *B. pertussis* may have occurred in signaling cascades activating T3SS and/or BteA effector protein expression during *in vivo* infection. Alternatively, or in combination, a functional divergence could originate in the level of effector specific activities as recently described by Bayram et al. (2020). The *Bp* BteA has only residual cytotoxic activity as compared to *Bb* BteA, and the deletion of a differing A503 residue from the wild type *Bp* BteA (Figure 5A) increased T3SS-mediated *B. pertussis* cytotoxicity. Besides, the mutant *B. pertussis bteA*ΔA503 exhibited a higher virulence in the mouse model of intranasal infection, while at a sublethal challenge dose it accounted for a reduced pathology in *B. pertussis*-infected mouse lungs. These data show that a more active BteAΔA503 was able to importantly intervene in the interactions of *B. pertussis* with the host defense and its action shaped the course and outcome of the infection (Bayram et al., 2020).

## Concluding Remarks and Outstanding Questions

Since 1906, when the founding member of the *Bordetella* genus, *B. pertussis*, was isolated by Bordet and Gengou from Bordet's son suffering from pertussis, our comprehension of the bordetellae species has come a long way. Nevertheless, many unknowns remain and dozens of outstanding questions can be formulated in the very specific area of research on the type III secretion in bordetellae. Perhaps the most important question is the role of T3SS activity in the pathophysiology of human pertussis. Hopefully, the availability of a non-human primate in which the human pathology of pertussis and transmission of the pathogen can be reproduced in olive baboons will yield an answer (Pinto and Merkel, 2017; Zimmerman et al., 2018). It remains to be determined if the variation in the T3SS contributed to *B. pertussis* evolution and pertussis pathogenesis. A better insight into the regulation of the T3SS expression and injectosome functionality is also needed. It remains unclear what are the host signals that *Bordetella* spp. respond to and whether these signals and/or signaling cascades are different for *B. pertussis* and *B. bronchiseptica*. We still do not understand by which mechanism do *B. pertussis* bacteria turn off the T3SS activity upon passage on laboratory media (Gaillard et al., 2011) and why despite the potent cytotoxicity in cultured cells the *in vivo B. bronchiseptica*-colonized respiratory epithelia shows no damage (Cotter and Miller, 1994; Panina et al., 2005). Future research should also focus on the understanding of the *Bordetella* injectosome that differs by the presence of the Bsp22 helical assembly and YscX-like and YscY-like proteins from the well-studied injectosomes of *Salmonella enterica* and *Shigella* spp. Last not least, we need to get a better comprehension of the molecular details behind the actions of *Bordetella* effector protein/s, their function and interplay with other virulence factors of classical *Bordetella* species.

## Author Contributions

The author confirms being the sole contributor of this work and has approved it for publication.

## Conflict of Interest

The author declares that the research was conducted in the absence of any commercial or financial relationships that could be construed as a potential conflict of interest.
